# A national survey on temporary and delayed abdominal closure in Norwegian hospitals

**DOI:** 10.1186/1757-7241-19-51

**Published:** 2011-09-14

**Authors:** Sigrid Groven, Pål A Næss, Erik Trondsen, Christine Gaarder

**Affiliations:** 1 Department of Traumatology, Oslo University Hospital Ullevaal, Oslo, Norway; 2 Department of Surgery, Vestre Viken HF, Drammen Hospital, Drammen, Norway; 3 Department of GI Surgery, Oslo University Hospital Ullevaal, Oslo, Norway

**Keywords:** temporary abdominal closure, damage control surgery, abdominal compartment syndrome, survey

## Abstract

**Introduction:**

Temporary abdominal closure (TAC) is included in most published damage control (DC) and abdominal compartment (ACS) protocols. TAC is associated with a range of complications and the optimal method remains to be defined. The aim of the present study was to describe the experience regarding TAC after trauma and ACS in all acute care hospitals in a sparsely populated country with long transportation distances.

**Material and methods:**

A questionnaire was sent to all 50 Norwegian hospitals with acute care general surgical services.

**Results:**

The response rate was 88%. A very limited number of hospitals had treated more than one trauma patient with TAC (5%) or one patient with ACS (14%) on average per year. Most hospitals preferred vacuum assisted techniques, but few reported having formal protocols for TAC or ACS. Although most hospitals would refer patients with TAC to a trauma centre, more than 50% reported that they would perform a secondary reconstruction procedure themselves.

**Conclusion:**

This study shows that most Norwegian hospitals have limited experience with TAC and ACS. However, the long distances between hospitals mandate all acute care hospitals to implement formal treatment protocols including monitoring of IAP, diagnosing and decompression of ACS, and the use of TAC. Assuming experience leads to better care, the subsequent treatment of these patients might benefit from centralization to one or a few regional centers.

## Introduction

Damage control techniques as well as prevention and treatment of abdominal compartment syndrome (ACS) includes the use of temporary abdominal closure (TAC), resulting in the clinical challenges of open abdomen-related morbidity. A wide variety of TAC techniques exists, including commercial or improvised vacuum-assisted closure, permanent or absorbable prosthetic mesh insertion, Bogota bag, or strategies using native tissue only, leaving the optimal TAC yet to be defined. There is no standardization of terminology or accepted guidelines for when to leave the abdomen open, and controversy exists among surgeons as to which of the different options for TAC to select [1].

All TAC techniques are associated with a range of complications, as surgical site infections, sepsis, prolonged stay in the intensive care unit (ICU), enteroatmospheric fistulas and large hernias [2-9]. Follow-up of patients with an open abdomen demands multidisciplinary teamwork. The optimal management of the open abdomen remains one of our major surgical challenges [1,10].

Only few published surveys address this complex patient group, showing absence of standardized approach, and a wide variation in clinical management [1,11].

Through a national survey, the aim of the present study was to describe the experience regarding TAC in the trauma context and in patients with ACS regardless of etiology in all acute care hospitals in a sparsely populated country with long transportation distances.

## Materials and methods

Norway is a sparsely populated country, covering 323.000 square kilometers with a population of 4.7 million people. There is a total of 50 hospitals with acute care surgical facilities, resulting in low patient volumes and long transportation distances for many of the hospitals.

**Figure 1:**
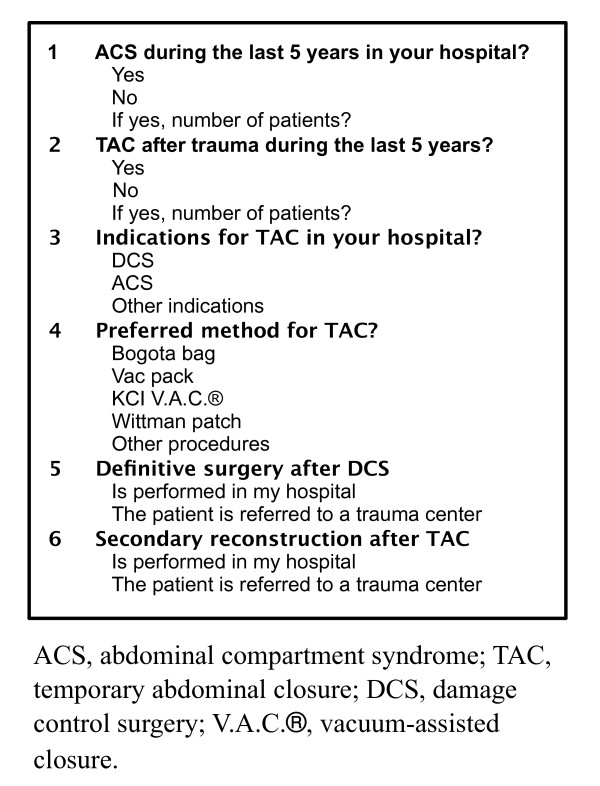
**Initial questionnaire**.

**Figure 2:**
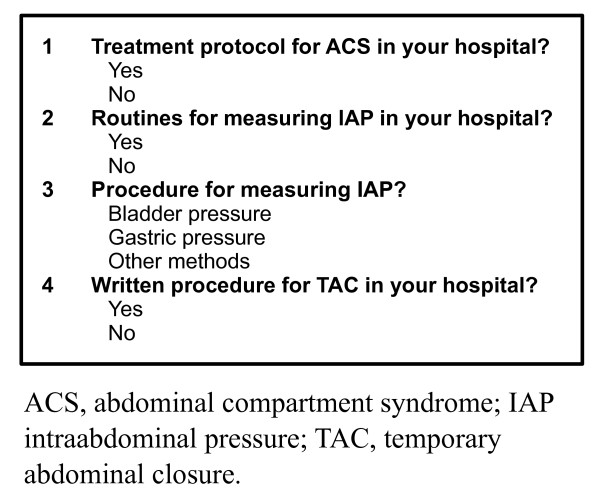
**Follow-up internet-based questionnaire**.

A questionnaire (Figure 1) was in March 2009 sent to one attending surgeon in every general/gastrointestinal (GI) surgical department in all hospitals with acute care surgical facilities in order to assess the experience with TAC in the trauma context and in patients with ACS regardless of etiology over the last five years. Questionnaires were coded to maintain confidentiality and to track hospitals having responded for the purpose of avoiding unnecessary renotification. To increase the response rate a renotification was sent after two months. A follow-up internet-based questionnaire (Figure 2) to assess protocols and routines in this field was sent to the same departments one year after the initial survey.

## Results

Completed questionnaires were received from 44 of the 50 hospitals including 4 out of 5 regional trauma centres, yielding a response rate of 88%. Twelve of the hospitals (27%) had treated trauma patients with TAC during the last five years, and only 2 of these hospitals had treated more than one patient on average per year.

Most hospitals reported that they would use well established techniques for TAC, with 25 hospitals preferring a modified Opsite^® ^sandwich technique (vacuum pack) [12] and 12 hospitals reporting that they would use the KCI V.A.C.^® ^(Kinetic Concepts Inc. International, San Antonio, TX, USA). Only 3 hospitals would use the Bogota bag, while 9 hospitals chose another, unspecified method. Several hospitals reported more than one type of procedure.

A total of 27 hospitals (61%) reported that they would refer patients with TAC after damage control surgery (DCS) to a trauma centre, while the rest would perform the definitive surgical treatment of the injury and closure of the abdomen themselves. If secondary reconstruction after TAC was indicated, only 21 of the 44 hospitals (48%) would have transferred the patient to a regional centre.

In addition to DCS, 23 of the hospitals (52%) reported ACS regardless of etiology as an indication for TAC. A total of 22 hospitals (50%) reported having treated patients with ACS, but only 6 hospitals had treated more than one patient on average per year.

The follow-up survey was conducted to describe existing protocols and routines for TAC, ACS and monitoring of intraabdominal pressure (IAP). Completed questionnaires were recieved from 31 of the 50 hospitals, yielding a response rate of 62%. Of these 31 hospitals, 24 (77%) reported having routines for measuring IAP in risk patients. Bladder pressure measurement was the only reported method. Formal protocols for treating ACS existed in only 10 hospitals, while 11 hospitals reported having formal protocols for TAC.

## Discussion

This national survey indicates that most surgical departments have limited experience with this complex patient group, with only 2 hospitals reporting having treated more than one trauma patient with TAC on average per year over the study period. Accordingly, only 6 hospitals reported having treated more than one patient with ACS on average per year, regardless of etiology. Our findings seem to be in agreement with Kirkpatrick et al. [1], showing no consensus nor standard methods for closure of the open abdomen among the members of Trauma Association of Canada. Karmali et al. [11] assessed the opinion of the same group of Canadian trauma surgeons while Mayberry et al. [13] assessed the opinion of members of the American Association for the Surgery of Trauma. Through description of physicians' response to various clinical scenarios, they revealed a widespread knowledge on ACS [13], while no particular procedure for TAC seemed to have gained general acceptance [11].

Addressing members of professional societies carries the inherent risk of getting several answers from some hospitals and none from others. In contrast to the above mentioned surveys, our study is the first to address all general surgical departments in a country regarding their experience with TAC and ACS, and achieving a high response rate.

An ideal TAC should cover and protect abdominal contents, manage excessive fluid, avoid damaging the fascia, minimize loss of domain, limit risk for complications and facilitate reoperation and closure [14]. The negative pressure techniques report low incidence of complications and high closure rates [3,4,7,14-16], and are recommended- at least in the initial phase- by the Open Abdomen Advisory Panel in 2009 [14]. Although only about one third of the hospitals in Norway state having standardized protocols for TAC, the current practice seems to be according to these recommendations.

Primary ACS in centres with appropriate level of awareness should now be extremely rare [10]. However, Kimball et al. [17] revealed that among members of the Society of Critical Care Medicine, 82,8% of the respondents had treated one or more patients during the last year. Tiwari et al. [18] did a survey of ICUs in the United Kingdom revealing that 96,9% of the teaching hospitals and 72,6% of the district general hospitals had seen ACS. In our study 50% of the hospitals reported having treated patients with ACS during the last five years, but only 13% had treated more than one patient per year on average. Ravishankar et al. [19] showed that many intensive care units in the United Kingdom never measure IAP. In our follow-up survey, 77% of the hospitals reported having routines for measuring IAP. However, our study does not assess whether the correct risk patients are identified, with the potential of giving us an underestimate of the actual incidence.

The follow up of patients with TAC is complex and requires extensive multidisplinary teamwork and experience [1,11,14]. After damage control resuscitation and application of TAC, the patient proceeds through phases with different management goals. The optimal final aim is to achieve definitive abdominal closure within the initial hospitalization, and with as few complications as possible. Norway is a sparsely populated country with long transportation distances much like other rural areas worldwide, mandating hospitals providing acute care and initial trauma care to have procedures for damage control and TAC. Given the low patient volume and limited experience revealed in the present survey these patients might benefit from referral to a centre with surgical experience and necessary critical care resources, to optimize further treatment.

A proportion of the patients will have fascial defects that cannot be closed during the initial hospitalization. When secondary reconstruction is indicated, more than half of the respondents in our study would have performed the surgery locally- even though their experience is limited. For some of the hospitals it remains a hypothetical problem, since more than 70% reported not having treated a trauma patient with TAC during the last five years.

The study has several additional limitations. It is retrospective and subject to recall bias due to the lack of trauma and critical care registries in most hospitals. ICUs in Norway are run by anaesthesiology trained intensivists. However, surgeons are involved in the care of their patients in ICU and should be aware of patients at risk of IAH and ACS. The questionnaires did not explore the use of TAC as part of the strategy to avoid ACS in other patient categories than trauma, hence the number of patients treated with TAC in each hospital might be underestimated. Finally, the surgeons' subjective response might not correspond to the hospitals' current clinical practice.

## Conclusion

This study shows that most Norwegian hospitals have limited experience with TAC and ACS. However, the long distances between hospitals mandate all acute care hospitals to implement formal treatment protocols including monitoring of IAP, diagnosing and decompression of ACS, and the use of TAC. Assuming experience leads to better care, the subsequent treatment of these patients might benefit from centralization to one or a few regional centers.

## Competing interests

The authors declare that they have no competing interests.

## Authors' contributions

SG, PAN and CG had the original idea for the study and developed the questionnaires. SG developed the database. Data were analyzed by all authors. All authors contributed in the preparation of the manuscript.

## References

[B1] Kirkpatrick AW Laupland KB Karmali S Bergeron E Stewart TC Findlay C Spill your guts! Perceptions of Trauma Association of Canada member surgeons regarding the open abdomen and the abdominal compartment syndrome J Trauma 2006 60 2 279 286 10.1097/01.ta.0000205638.26798.dc 16508483

[B2] Nagy KK Fildes JJ Mahr C Roberts RR Krosner SM Joseph KT Experience with three prosthetic materials in temporary abdominal wall closure Am Surg 1996 62 5 331 335 8615556

[B3] Barker DE Kaufman HJ Smith LA Ciraulo DL Richart CL Burns RP Vacuum pack technique of temporary abdominal closure: a 7-year experience with 112 patients J Trauma 2000 48 2 201 206 10.1097/00005373-200002000-00001 10697075

[B4] Garner GB Ware DN Cocanour CS Duke JH McKinley BA Kozar RA Vacuum-assisted wound closure provides early fascial reapproximation in trauma patients with open abdomens Am J Surg 2001 182 6 630 638 10.1016/S0002-9610(01)00786-3 11839329

[B5] Miller RS Morris JA Jr Diaz JJ Jr Herring MB May AK Complications after 344 damage-control open celiotomies J Trauma 2005 59 6 1365 1371 10.1097/01.ta.0000196004.49422.af 16394910

[B6] Montalvo JA Acosta JA Rodriguez P Alejandro K Sarraga A Surgical complications and causes of death in trauma patients that require temporary abdominal closure Am Surg 2005 71 3 219 224 15869136 10.1177/000313480507100309

[B7] Becker HP Willms A Schwab R Small bowel fistulas and the open abdomen Scand J Surg 2007 96 4 263 271 18265852 10.1177/145749690709600402

[B8] Bee TK Croce MA Magnotti LJ Zarzaur BL Maish GO III Minard G Temporary abdominal closure techniques: a prospective randomized trial comparing polyglactin 910 mesh and vacuum-assisted closure J Trauma 2008 65 2 337 342 10.1097/TA.0b013e31817fa451 18695468

[B9] Fischer PE Fabian TC Magnotti LJ Schroeppel TJ Bee TK Maish GO III A ten-year review of enterocutaneous fistulas after laparotomy for trauma J Trauma 2009 67 5 924 928 10.1097/TA.0b013e3181ad5463 19901649

[B10] Balogh ZJ van WK Yoshino O Moore FA Postinjury abdominal compartment syndrome: are we winning the battle? World J Surg 2009 33 6 1134 1141 10.1007/s00268-009-0002-x 19343417

[B11] Karmali S Evans D Laupland KB Findlay C Ball CG Bergeron E To close or not to close, that is one of the questions? Perceptions of Trauma Association of Canada surgical members on the management of the open abdomen J Trauma 2006 60 2 287 293 10.1097/01.ta.0000203579.62446.75 16508484

[B12] Brock WB Barker DE Burns RP Temporary closure of open abdominal wounds: the vacuum pack Am Surg 1995 61 1 30 35 7832378

[B13] Mayberry JC Goldman RK Mullins RJ Brand DM Crass RA Trunkey DD Surveyed opinion of American trauma surgeons on the prevention of the abdominal compartment syndrome J Trauma 1999 47 3 509 513 10.1097/00005373-199909000-00012 10498305

[B14] Vargo D Richardson D Campell A Chang M Management of the Open Abdomen: From Initial Operation to Definitive Closure The American Surgeon 2009 75 11 S1 S22 19998714

[B15] Gaarder C Naess PA Schwab CW Bjornbeth BA Buanes T Pillgram-Larsen J Vacuum pack technique-a good method for temporal abdominal closure Tidsskr Nor Laegeforen 2004 124 21 2760 2762 15534669

[B16] Barker DE Green JM Maxwell RA Smith PW Mejia VA Dart BW Experience with vacuum-pack temporary abdominal wound closure in 258 trauma and general and vascular surgical patients J Am Coll Surg 2007 204 5 784 792 10.1016/j.jamcollsurg.2006.12.039 17481484

[B17] Kimball EJ Rollins MD Mone MC Hansen HJ Baraghoshi GK Johnston C Survey of intensive care physicians on the recognition and management of intra-abdominal hypertension and abdominal compartment syndrome Crit Care Med 2006 34 9 2340 2348 10.1097/01.CCM.0000233874.88032.1C 16878034

[B18] Tiwari A Myint F Hamilton G Recognition and management of abdominal compartment syndrome in the United Kingdom Intensive Care Med 2006 32 6 906 909 10.1007/s00134-006-0106-9 16601965

[B19] Ravishankar N Hunter J Measurement of intra-abdominal pressure in intensive care units in the United Kingdom: a national postal questionnaire study Br J Anaesth 2005 94 6 763 766 10.1093/bja/aei117 15764629

